# Lycium Barbarum polysaccharide protects HaCaT cells from PM2.5-induced apoptosis via inhibiting oxidative stress, ER stress and autophagy

**DOI:** 10.1080/13510002.2022.2036507

**Published:** 2022-02-07

**Authors:** Sen Zhu, Xuan Li, Bingrong Dang, Fen Wu, Chunming Wang, Changjun Lin

**Affiliations:** a School of Life Sciences, Lanzhou University, Lanzhou, People’s Republic of China; b Lanzhou University Second Hospital, Lanzhou, People’s Republic of China; c Institute of Modern Physics, Chinese Academy of Sciences, Lanzhou, People’s Republic of China

**Keywords:** PM2.5, Lycium barbarum polysaccharide (LBP), antioxidant, oxidative damage, endoplasmic reticulum (ER) stress, autophagy, apoptosis, mitochondrial damage

## Abstract

**Objectives:** Lycium barbarum polysaccharide (LBP) is a natural polysaccharide extracted from Lycium barbarum that has anti-inflammatory, anti-apoptotic and anti-aging effects, and plays a role in the prevention and treatment of various diseases. In this study, we investigated the therapeutic effect of LBP on particulate matter 2.5 (PM2.5)-induced skin damage.

**Methods:** Cell viability was analyzed by MTT and LDH assays. Apoptosis was analyzed by Annexin V-FITC/PI staining. Oxidative stress/damage were assessed by intracellular ROS levels, MDA content and SOD activity. The intracellular protein expression was analyzed by Western blot. Mitochondrial damage was assayed by mitochondrial membrane potential with JC-1 probe. LC3-GFP adenovirus was transfected into HaCaT cells to analyze intracellular autophagosome levels.

**Results:** In PM2.5-treated HaCaT cells, LBP pretreatment reduced PM2.5-induced cytotoxicity, ameliorated cell morphology and reduced cell apoptosis. LBP also inhibited the expression levels of GRP78 and CHOP, reduced the conversion of LC3I to LC3II, inhibited Bax protein and activated Bcl-2 protein. Furthermore, LBP inhibited PM2.5-induced mitochondrial autophagy (mitophagy) and mitochondrial damage. PM2.5-induced autophagy was regulated by endoplasmic reticulum (ER) stress.

**Conclusion:** LBP protects skin cells from PM2.5-induced cytotoxicity by regulating the oxidative stress-ER stress-autophagy-apoptosis signaling axis, revealing that LBP has a great potential for the skin protection.

## GRAPHICAL ABSTRACT

**Figure:**
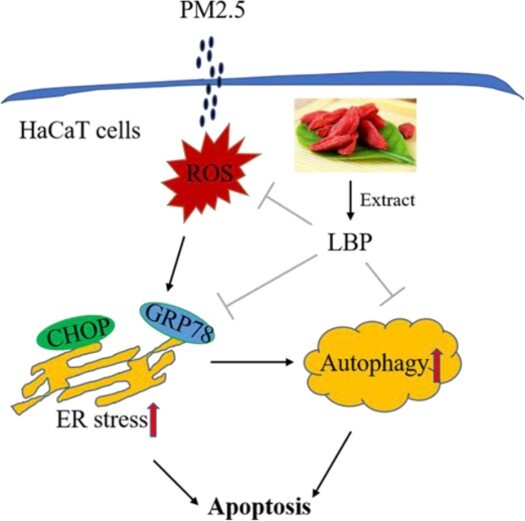

## 1. Introduction

Particulate matter 2.5 (PM2.5) is particles smaller than 2.5 microns in diameter, recognized as the main cause of air pollution and mainly derived from the combustion of coal and the exhaust of automobile. Many studies have shown that PM2.5 has many effects on human health, including provoking respiratory diseases, asthma, cardiovascular and cerebrovascular diseases [1–3]. Not only that, with air pollution getting more serious, the incidence of skin diseases has gradually increased [4]. As the largest tissue in the human body, skin is often exposed to air pollution, and it is susceptible to toxicity induced by PM2.5, including skin age, inflammation, DNA damage and carcinogenic effects [5,6].

In the study of Li et al., when HaCaT cells (immortalized non-tumorigenic human keratinocytes) are exposed to PM2.5, cell viability decreased and inflammation was induced [7]. Hu et al. also showed that PM2.5 could induce oxidative stress and oxidative damage, as well as apoptosis in HaCaT cells [8]. Immediately afterwards, Piao et al. demonstrated that when HaCaT cells were exposed to PM2.5, intracellular reactive oxygen species (ROS) were increased, and subsequently the increased ROS induced endoplasmic reticulum (ER) stress, mitochondrial damage and autophagy, finally leading to cell apoptosis [9]. These studies reveal the potential mechanism of PM2.5 causing skin damage and degeneration, which provides us with a target for the prevention and treatment of skin damage.

The ER is the main place for protein folding and processing, which maintains cell homeostasis and function [10]. When cells are stimulated by interference factors, the ER stress response will be activated to assist cells in resisting unfavorable factors [11]. However, excessive stimulation can induce the expression of apoptotic transcription factor CHOP and trigger cell apoptosis [12]. Autophagy is another cell metabolism mechanism that can clean up damaged organelles and recycle metabolic components [13]. Autophagy is a mechanism of cell self-protection, but abundant evidence also indicates that autophagy can induce apoptosis and intensify cytotoxicity [14].

Lycium barbarum polysaccharide (LBP) is the main active component of Lycium barbarum, and it has been widely reported to have anti-aging, anti-oxidant, anti-apoptotic and anti-inflammatory effects [15–17]. LBP has been revealed to have a great potential in the prevention and treatment of various diseases, including anti-adolescent depression, neuroprotection, retinal protection, and protection against heart failure [18–21]. In recent years, it has been reported that LBP can regulate the level of intracellular oxidative stress by regulating the Nrf2 signal to protect HaCaT cells and human skin fibroblast (HSF) cells from ultraviolet (UV) radiation-induced damage [17,22]. However, the protective effect of LBP on PM2.5-induced damage remains unclear. In this study, the protective effect of LBP on PM2.5-induced damage in HaCaT cells and its underlying mechanism were investigated.

## 2. Materials and methods

### 2.1. Materials

Particulate matter in the air was sampled by an air sampler (Thermo Anderson G-2.5, Thermo Scientific, USA) in Lanzhou, Gansu province for 3 months. After the collection was completed, PM2.5 was obtained by filtering through an ultra-fine glass fiber net. After that, PM2.5 was sterilized, then freeze-dried and concentrated and stored at −80°C for later use. HaCaT cell line was purchased from the Cell Bank of the Chinese Academy of Sciences (Shanghai, China). LBP was purchased from Natural Field Bio-Technique Co., Ltd (Shaanxi, China). DMEM medium was obtained from Hyclone (Beijing, China). Fetal Bovine Serum (FBS) was purchased from Si-Ji-Qing Biotechnology (Hangzhou, China). 2’,7’-Dichlorofluorescin diacetate was purchased from Sigma-Aldrich (St. Louis, MO, USA). Anti-LC3B and anti-P62 antibodies were purchased from Cell Signaling Technology (Danvers, MA, USA). Anti-GRP78 and anti-CHOP antibodies were purchased from Beyotime (Shanghai, China). Ad-GFP-LC3B adenovirus, Annexin V-FITC/PI apoptosis detection kit, Mitochondrial membrane potential detection kit (JC-1) and the Ca^2+^ probe Fluo-4 AM were also purchased form Beyotime (Shanghai, China).

### 2.2. Cell culture and treatment

HaCaT cells were cultured in DMEM high glucose medium supplemented with 10% FBS, 100 U/ml penicillin, and 100 mg/ml streptomycin at 37°C in air containing 5% CO_2_. HaCaT cells were pretreated with LBP (or 3-MA or 4-PBA) for 2 h and incubated with PM2.5 for 24 h, and then the cells were collected for subsequent experiments.

### 2.3. Cell viability analysis

The viability of HaCaT cells was detected by MTT method, consistent with our previous research [23]. In addition, lactate dehydrogenase (LDH) release was also used to analyze the cytotoxicity, according to the manufacturer's instructions (Nanjing Jiancheng Bioengineering Institute, Nanjing, China). The LDH release was detected at 490 nm wavelength by a microplate reader.

### 2.4. Apoptosis assay with Annexin V-FITC/PI by a confocal microscope

HaCaT cells (1 × 10^5^ cells/well) were seeded in 6-well plates for 24 h. Then cells were pretreated with or without LBP (or 3-MA) for 2 h, and subsequently treated with or without PM2.5 (100 μg/ml) for another 24 h. Apoptosis was detected by a confocal microscope (Nikon, A1R+ Ti2-E), using an Annexin V- FITC/PI Apoptosis Kit.

### 2.5. Measurement of ROS production

HaCaT cells (1 × 105 cells/well) were seeded in 6-well plates for 24 h. Cells were pretreated with or without LBP for 2 h, and then co-incubated with or without PM2.5 for 24 h. Subsequently, fluorescent probes DCFH-DA and DHE-labeled intracellular ROS, with Hoches33342-counterstained the nucleus, were observed under a fluorescent microscope.

### 2.6. Detection of MDA levels

HaCaT cells (3 × 10^5^ cells/well) were seeded in 6-well plates for 24 h. Then cells were pretreated with or without LBP for 2 h, and subsequently treated with or without PM2.5 (100 μg/ml) for another 24 h. MDA content was detected with the kit provided by the Nanjing Jiancheng Bioengineering Institute (Nanjing, China), according to the manufacturer's instructions. In brief, MDA content is determined by its reaction with thiobarbituric acid (TBA) to form a colorimetric product, which can be detected at the 532 nm wavelength.

### 2.7. Detection of SOD activity

HaCaT cells (3 × 10^5^ cells/well) were seeded in 6-well plates for 24 h. Then cells were pretreated with or without LBP for 2 h, and subsequently treated with or without PM2.5 (100 μg/ml) for another 24 h. The SOD activity in HaCaT cells was detected by the kit provided by Nanjing Jiancheng Bioengineering Institute (Nanjing, China), according to the manufacturer's instructions. The intracellular SOD activity was detected at a wavelength of 450 nm.

### 2.8. Immunofluorescence detection

In brief, HaCaT cells were treated with LBP and PM2.5, and then washed three times with PBS and fixed with 4% paraformaldehyde at 25°C for 15 min. After cell fixation, incubated with primary antibody overnight at 4°C, and then washed three times with PBST and incubated with fluorescent secondary antibody for 1 h. After that, with DAPI counterstained-cell nucleus, the samples were observed by a confocal microscope (Nikon, A1R+ Ti2-E).

### 2.9. Western blot assay

HaCaT cells were pretreated with or without LBP (or 4-PBA) for 2 h and then treated with or without PM2.5 (100 μg/ml) for 24 h. Cells were collected and then lysed on ice using RIPA lysate. One percent of PMSF was added into lysate to prevent protein degradation. Further operation is consistent with our previous research [23].

### 2.10. Detection of intracellular Ca^2+^ levels

HaCaT cells were pretreated with LBP for 2 h, and then incubated with PM2.5 for 24 h. After cells were washed with PBS three times, the Ca^2+^ probe Fluo-4 AM was added and co-incubated in the dark for 15 min. The intracellular Ca^2+^ level was detected by a fluorescent microscope.

### 2.11. Measurement of autophagy levels

To measure autophagic vesicles, HaCaT cells were transfected with GFP-LC3 adenoviruses at a 20 multiplicity of infection (MOI). After cultured for 24 h, HaCaT cells were pretreated with LBP (or 4-PBA) for 2 h and then were treated with PM2.5 for 24 h. Furthermore, Mito tracker Red was incubated with HaCaT cells for 20 min to detect mitophagy. Then the fluorescent signals were detected by a confocal microscopy (Nikon, A1R+ Ti2-E).

### 2.12. Mitochondrial damage analysis

The decrease of mitochondrial membrane potential is an important marker of mitochondrial damage. HaCaT cells were pretreated with 3-MA or LBP for 2 h, and then incubated with PM2.5 for 24 h. After washed with PBS three times, cells were incubated with JC-1 probe in darkness for 30 min, and then mitochondrial membrane potential was detected by a confocal microscopy. When the mitochondrial membrane potential is normal, JC-1 is at the state of aggregates (red fluorescence); when the mitochondrial membrane potential is decreased, JC-1 can be de-aggregated into monomers (green fluorescence), indicating mitochondrial damage.

### 2.13. Statistical analysis

Data were collected from at least three independent experiments. Statistical analysis was performed as the mean ± standard deviation (SD). Data analysis was expressed using Prism 9.0 software (GraphPad Software) or Origin 8.0 software. The data were analyzed using student's t-test. Differences with *p *< 0.05 were considered statistically significant.

## 3. Results

### 3.1. LBP reduces PM2.5-triggered apoptosis and increases cell survival

**Figure 1.**
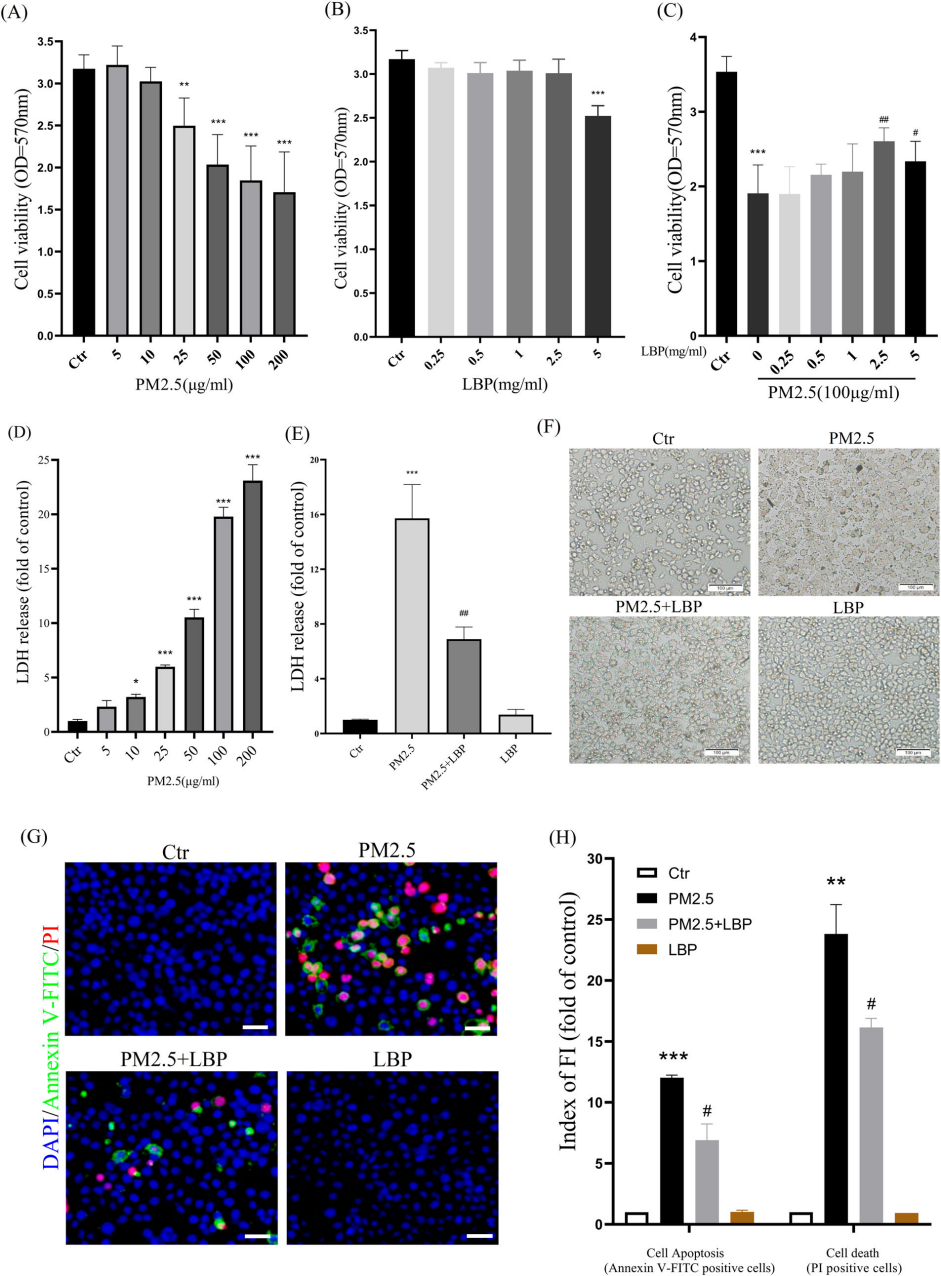
LBP reduces PM2.5-triggered apoptosis and increases cell survival. (A) MTT assay was used to detect the cytotoxicity of PM2.5 in HaCaT cells. (B) MTT assay was used to detect the effects of LBP on cell survival. (C) Cell viability was analyzed by MTT assay. HaCaT cells were pretreated with different concentrations of LBP and then co-incubated with PM2.5 for 24 h. (D) LDH assay was used to detect PM2.5-induced cytotoxicity in HaCaT cells. (E) Cell cytotoxicity was analyzed by LDH assay. HaCaT cells were pretreated with 2.5 mg/ml LBP and then co-incubated with PM2.5 for 24 h. (F) Cell morphology was analyzed by an inverted fluorescent microscope. Scale bar = 100 μm. (G) Apoptosis was analyzed with Annexin V-FITC, PI and DAPI staining, and observed under a fluorescent microscope. Scale bar = 50 μm. (H) Statistics of Annexin V-FITC and PI positive cells. Values are mean ± SD. *p < 0.05, **p < 0. 01, ***p < 0.001 versus the control group; #*p *< 0.05, ##*p *< 0.01, ###*p *< 0.001 versus the PM2.5 treatment alone group.

HaCaT cells were pretreated with LBP (0, 0.25, 0.5, 1, 2.5, 5 mg/ml) for 2 h and then exposed to PM2.5 (100 μg/ml) for 24 h. After that, we evaluated the protective effect of LBP on PM2.5-induced cytotoxicity by MTT and LDH assay. The results showed that PM2.5 induced cell proliferation inhibition and led to cell death, while LBP (2.5 or 5 mg/ml) pretreatment alleviated PM2.5-induced cytotoxicity (Figure 1(A) and 1(C)-1(E)). Since 5 mg/ml LBP was slightly toxic to HaCaT cells, we chose 2.5 mg/ml LBP as a follow-up study (Figure 1(B)). Moreover, the protective effect of LBP on HaCaT was evaluated by cell morphology. The results showed that LBP significantly ameliorated cell morphological damage induced by PM2.5 (Figure 1(F)). In addition, PM2.5-induced HaCaT apoptosis is an important factor that aggravates cytotoxicity and leads to cell damage. Therefore, we further used Annexin V-FITC and PI staining to evaluate the effect of LBP on cell apoptosis. The results showed that LBP significantly reduced PM2.5-induced apoptosis (Annexin V-FITC positive cells) and cell death (PI positive cells) (Figure 1(G) and 1(H)). The above evidence indicates that LBP alleviates PM2.5-induced HaCaT cytotoxicity, which may be through its anti-apoptotic effect.

### 3.2. LBP alleviates PM2.5-induced oxidative stress and oxidative damage in HaCaT cells

**Figure 2.**
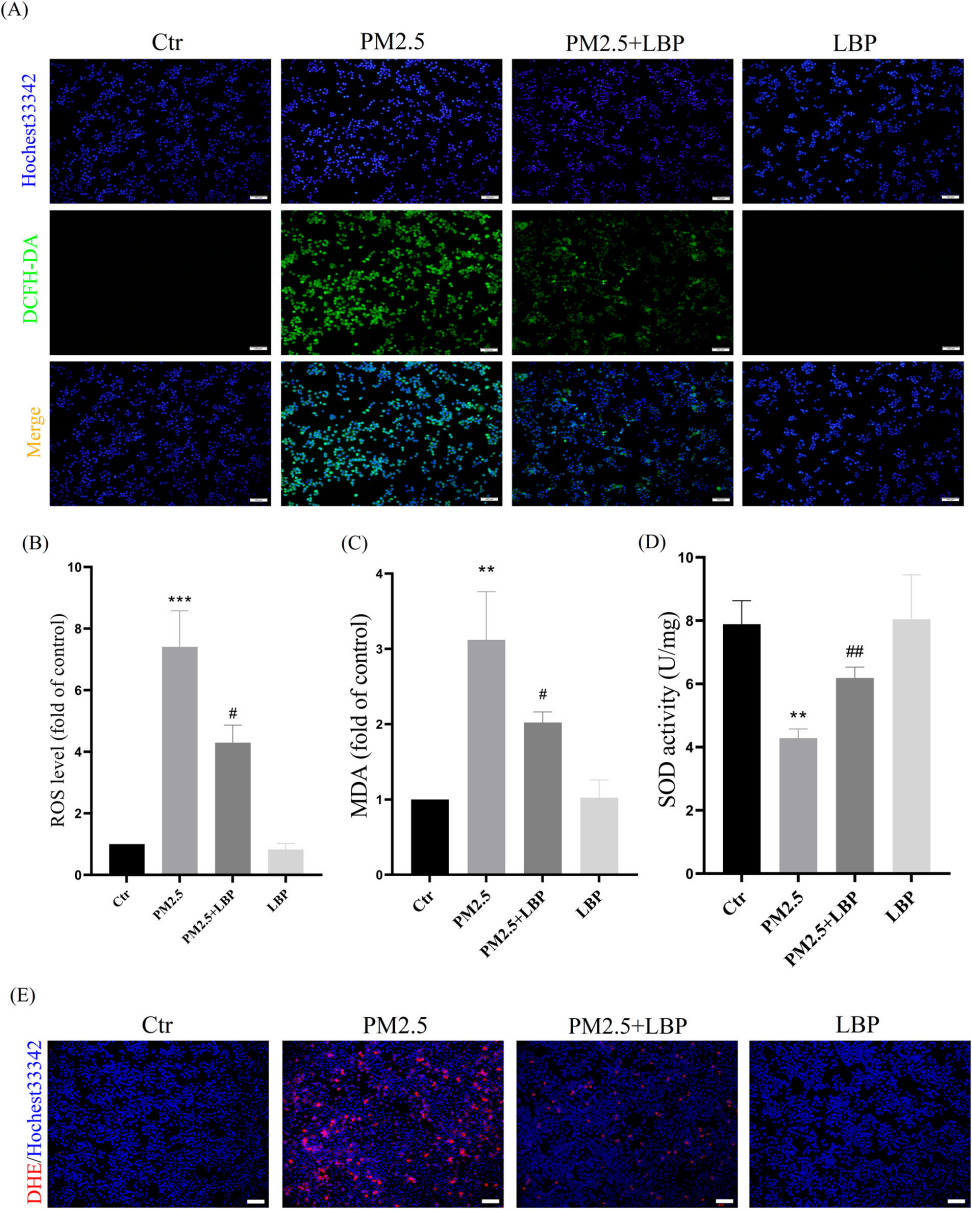
LBP alleviates PM2.5-induced oxidative stress and oxidative damage in HaCaT cells. (A) Cells were double-stained with Hochest33342 and DCFH-DA, and intracellular ROS levels were observed by a fluorescent microscopy. Scale bar = 100 μm. (B) Statistics of intracellular ROS level, stained with DCFH-DA. The intracellular MDA level (C) and SOD activity (D) were detected by the corresponding assay kits. (E) Cells were double-stained with Hochest33342 and DHE, and intracellular ROS levels were observed by a fluorescent microscopy. Scale bar = 100 μm. Values are mean ± SD. *p < 0.05, **p < 0. 01, ***p < 0.001 versus the control group; #*p *< 0.05, ##*p *< 0.01, ###*p *< 0.001 versus the PM2.5 treatment alone group.

We further studied the effect of LBP on PM2.5-induced oxidative stress and oxidative damage in HaCaT cells. HaCaT cells were pretreated with LBP (2.5 mg/ml) for 2 h, and then incubated with PM2.5 for 24 h. Intracellular ROS were labeled by DCFH-DA and DHE, and observed with a fluorescent microscope. The results showed that LBP significantly inhibited the increase of intracellular ROS (Figure 2(A), 2(B) and 2(E)). In addition, we evaluated the effect of LBP on PM2.5-induced oxidative damage and cellular antioxidant capacity through detecting MDA level and SOD activity. We found that LBP could alleviate PM2.5-induced MDA production (Figure 2(C)) and restore SOD activity (Figure 2(D)). All these results indicate that LBP can protect against PM2.5-induced oxidative stress and oxidative damage in HaCaT cells.

### 3.3. LBP inhibits PM2.5-induced ER stress and CHOP expression in HaCaT cells

**Figure 3.**
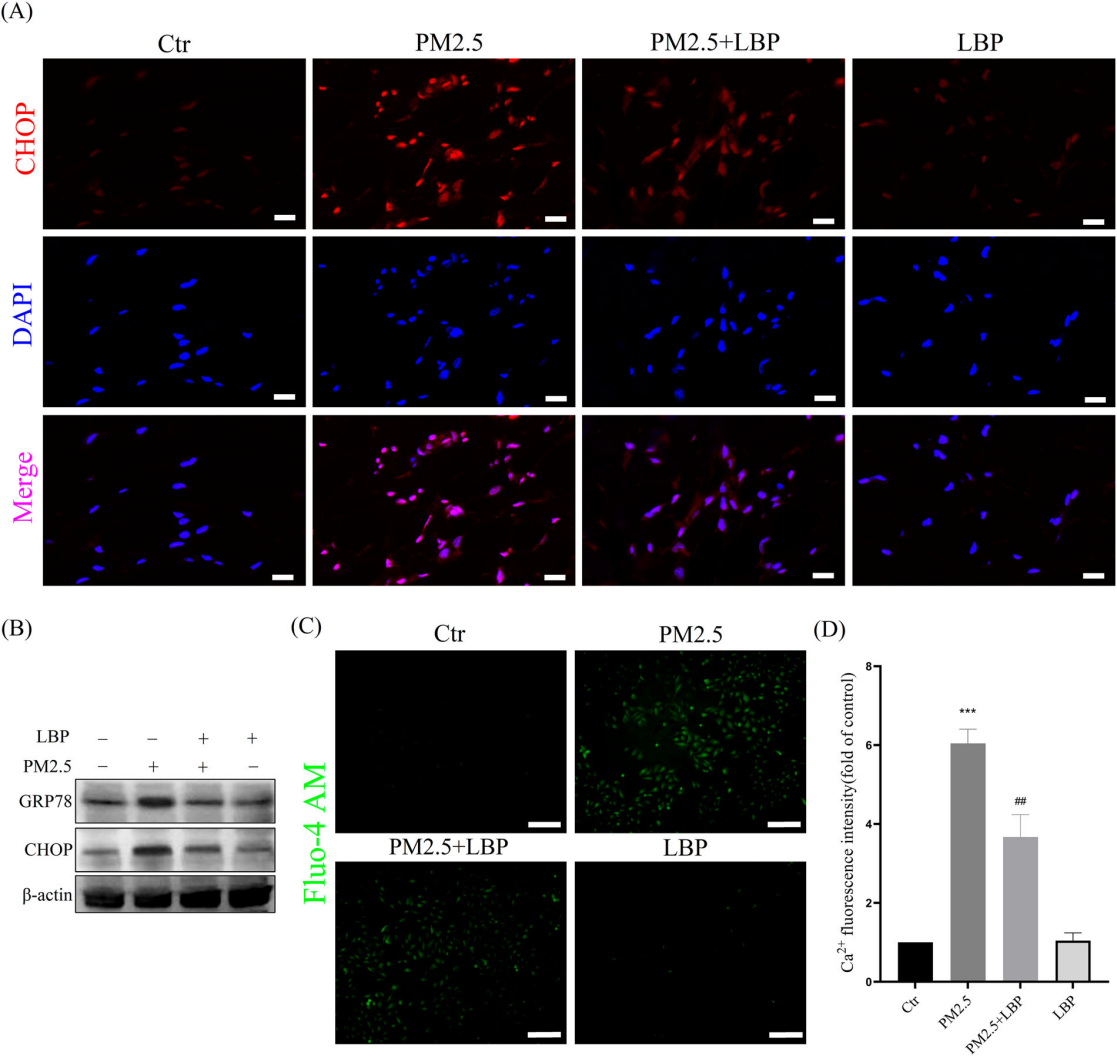
LBP inhibits PM2.5-induced ER stress and CHOP expression in HaCaT cells. (A) Immunofluorescence was used to detect the expression of CHOP in HaCaT cells. Scale bar = 20 μm. (B) Western blot assay was used to detect the protein levels of GRP78 and CHOP. (C) Intracellular Ca^2+^ was labeled with Fluo-4 AM and analyzed by a fluorescent microscope. Scale bar = 100 μm. (D) Statistics of intracellular Ca^2+^ level. Values are mean ± SD. *p < 0. 05 versus the control group; # *p *< 0.05 versus the PM2.5 treatment alone group.

Piao et al. proved that ROS-mediated ER stress-CHOP apoptotic signal was involved in PM2.5-induced apoptosis in HaCaT cells [9]. Recently, the study by Kim et al. also confirmed that by inhibiting the ER stress-CHOP apoptotic signal, PM2.5-induced apoptosis and toxicity could be alleviated [24]. Therefore, we further studied the effect of LBP on PM2.5-induced ER stress-CHOP signal. The results showed that LBP significantly inhibited PM2.5-induced GRP78 and CHOP expression, and reduced the accumulation of CHOP in the nucleus (Figure 3(A) and 3(B)). In addition, ER is the largest Ca^2+^ storage organelle, and ER stress can cause intracellular Ca^2+^ imbalance. In this study, the calcium probe Fluo-4 AM was used to study the regulation of LBP on intracellular Ca^2+^ homeostasis. The results showed that PM2.5 induced the imbalance of intracellular Ca^2+^, while LBP maintained the homeostasis of intracellular Ca^2+^ (Figure 3(C) and 3(D)). Our evidence suggests that LBP inhibits PM2.5-induced ER stress response.

### 3.4. ER stress-CHOP apoptosis signal is involved in PM2.5-triggered apoptosis and affects autophagy

**Figure 4.**
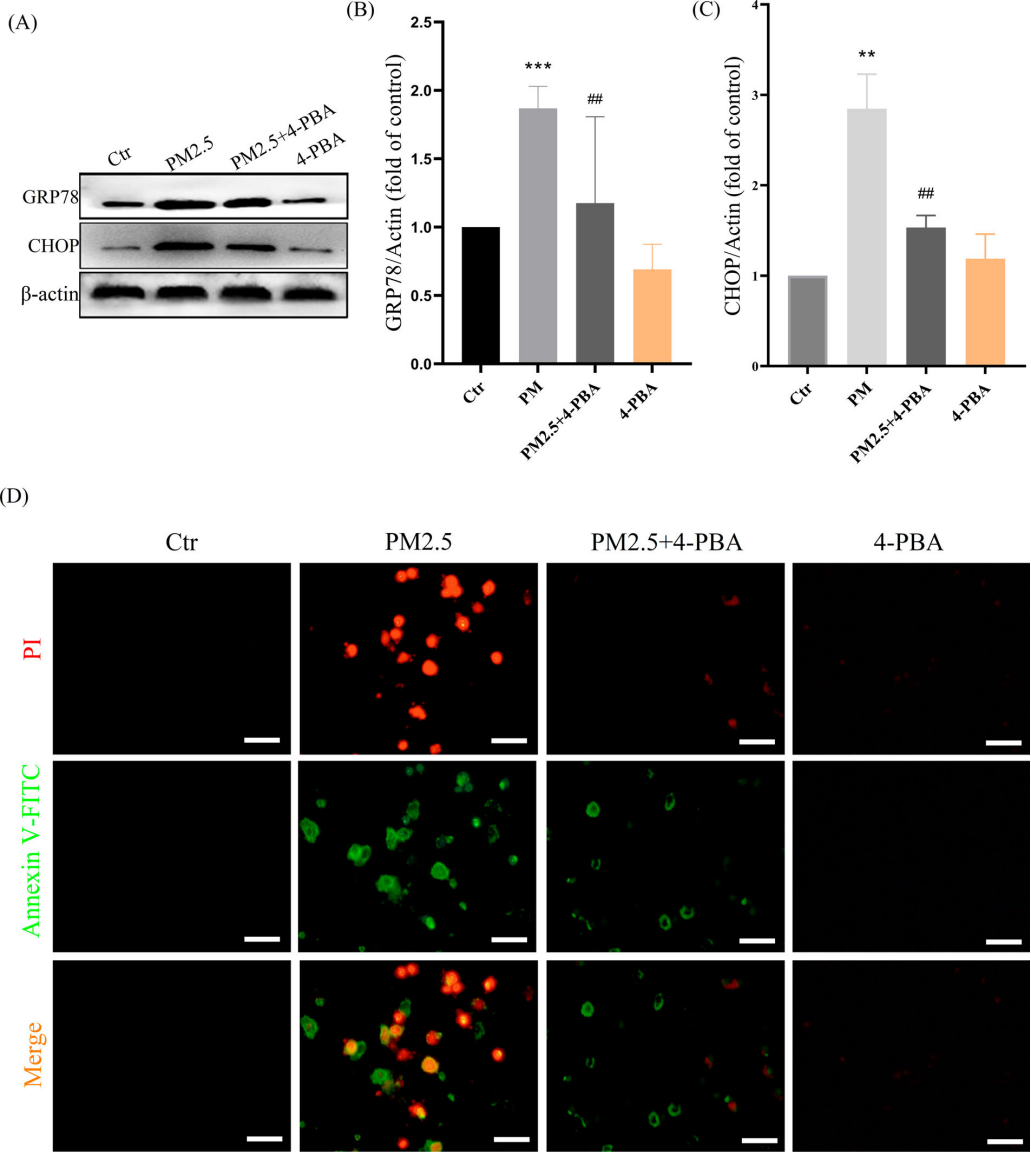
ER stress-CHOP apoptotic signal is involved in PM2.5-triggered apoptosis. (A) HaCaT cells were pretreated with 4-PBA (3 mM) for 2 h and then treated with PM2.5 for 24 h. Western blot was used to detect the expression levels of GRP78 and CHOP. (B) Statistics of GRP78 protein alteration. (C) Statistics of CHOP protein alteration. The gray levels were analyzed by the Image J software. (D) Apoptosis was analyzed with Annexin V-FITC and PI staining, and observed under a fluorescent microscope. Scale bar = 50 μm. Values are mean ± SD. **p < 0. 01, ***p < 0.001 versus the control group; ## *p *< 0.01 versus the PM2.5 treatment alone group.

**Figure 5.**
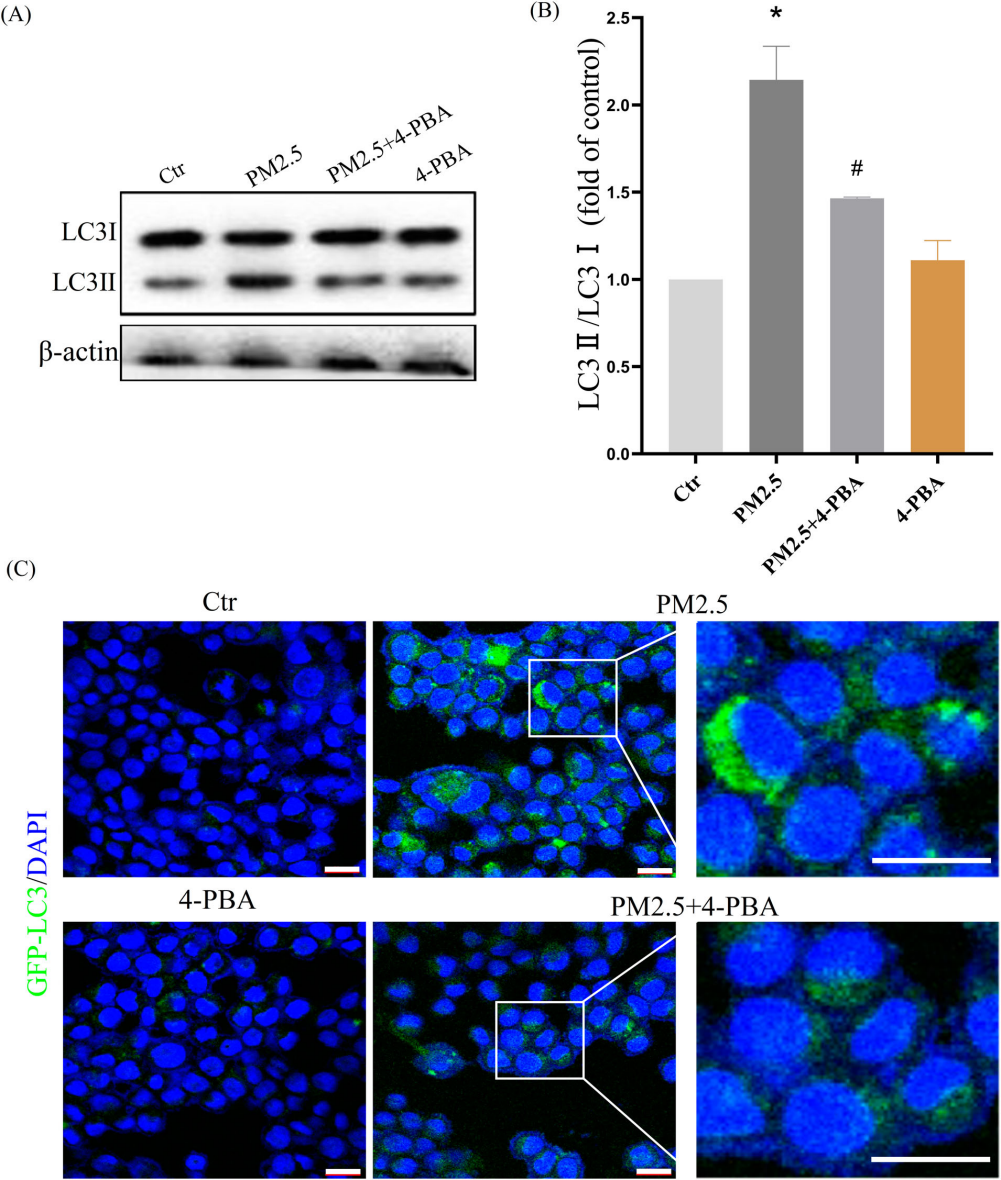
PM2.5-induced ER stress triggers autophagy in HaCaT cells. (A) Western blot assay of the autophagy marker proteins LC3II/LC3I;. (B) Statistics of LC3II/LC3I. (C) GFP-LC3 adenovirus was transfected into HaCaT cells. The fluorescent GFP-LC3 signal was used to detect autophagosomes under a confocal microscope. Scale bar = 20 μm. Values are mean ± SD. *p < 0.05 versus the control group; #*p *< 0.05 versus the PM2.5 treatment alone group.

To further confirm that LBP reduces PM2.5-triggered apoptosis by inhibiting the expression of CHOP, the ER stress inhibitor 4-PBA was used. HaCaT cells were pretreated with 4-PBA for 2 h, and then incubated with PM2.5 for 24 h. The results showed that PM2.5 induced the expression of GRP78 (1.87 ± 0.36, the control was set as ‘1’, the same as below) and CHOP (2.85 ± 0.39), while LBP pretreatment could significantly inhibit the expression of GRP78 (1.12 ± 0.66) and CHOP (1.53 ± 0.41) (Figure 4(A)-4(C)). Moreover, PM2.5-induced apoptosis and death were significantly reduced when CHOP was inhibited by 4-PBA (Figure 4(D)). The study by Piao et al. showed that in addition to inducing the ER stress response, PM2.5 also induced autophagy in HaCaT cells [9]. Interestingly, our results showed that when the ER stress response was inhibited by 4-PBA, the conversion of autophagy marker protein LC3I to LC3II was also inhibited (The value of LC3II/LC3I was reduced from 2.14 ± 0.24 to 1.46 ± 0.24) (Figure 5(A) and 5(B)). Furthermore, we transfected HaCaT cells with GFP-LC3 adenovirus to analyze autophagy vesicles. The results showed that 4-PBA inhibited the production of PM2.5-triggered autophagosome (Figure 5(C)). Our evidence demonstrates that PM2.5-induced ER stress signaling is involved in PM2.5-induced apoptosis in HaCaT cells and affects autophagy.

### 3.5. LBP inhibits autophagy triggered by PM2.5 to further reduce cytotoxicity

**Figure 6.**
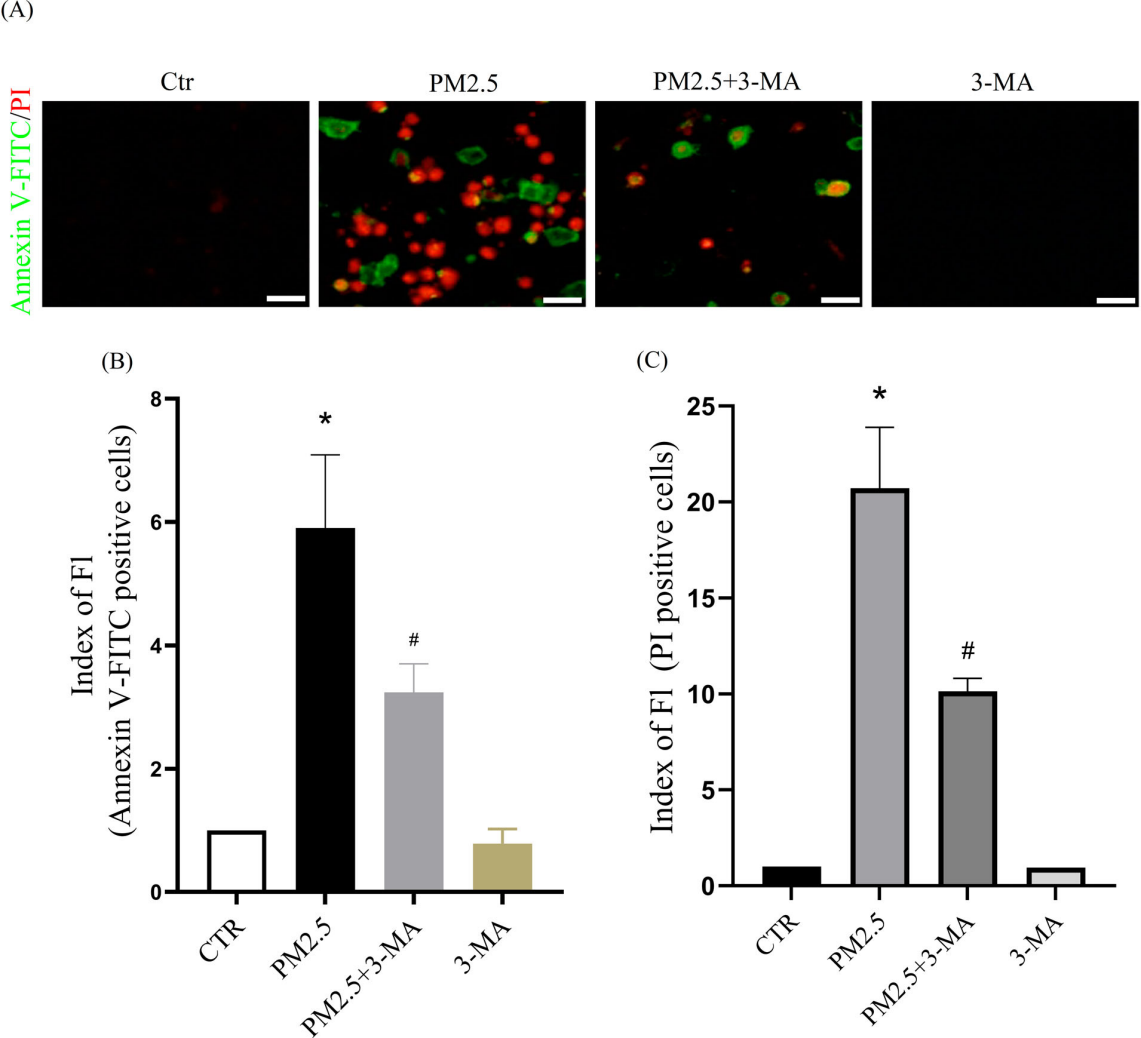
The autophagy inhibitor 3-MA reduces PM2.5-induced apoptosis and death. (A) Apoptosis was analyzed with Annexin V-FITC and PI staining, and observed under a fluorescent microscope. Scale bar = 50 μm. (B) Statistics of Annexin V-FITC positive cells. (C) Statistics of PI positive cells. Values are mean ± SD. *p < 0.05 versus the control group; #*p *< 0.05 versus the PM2.5 treatment alone group.

**Figure 7.**
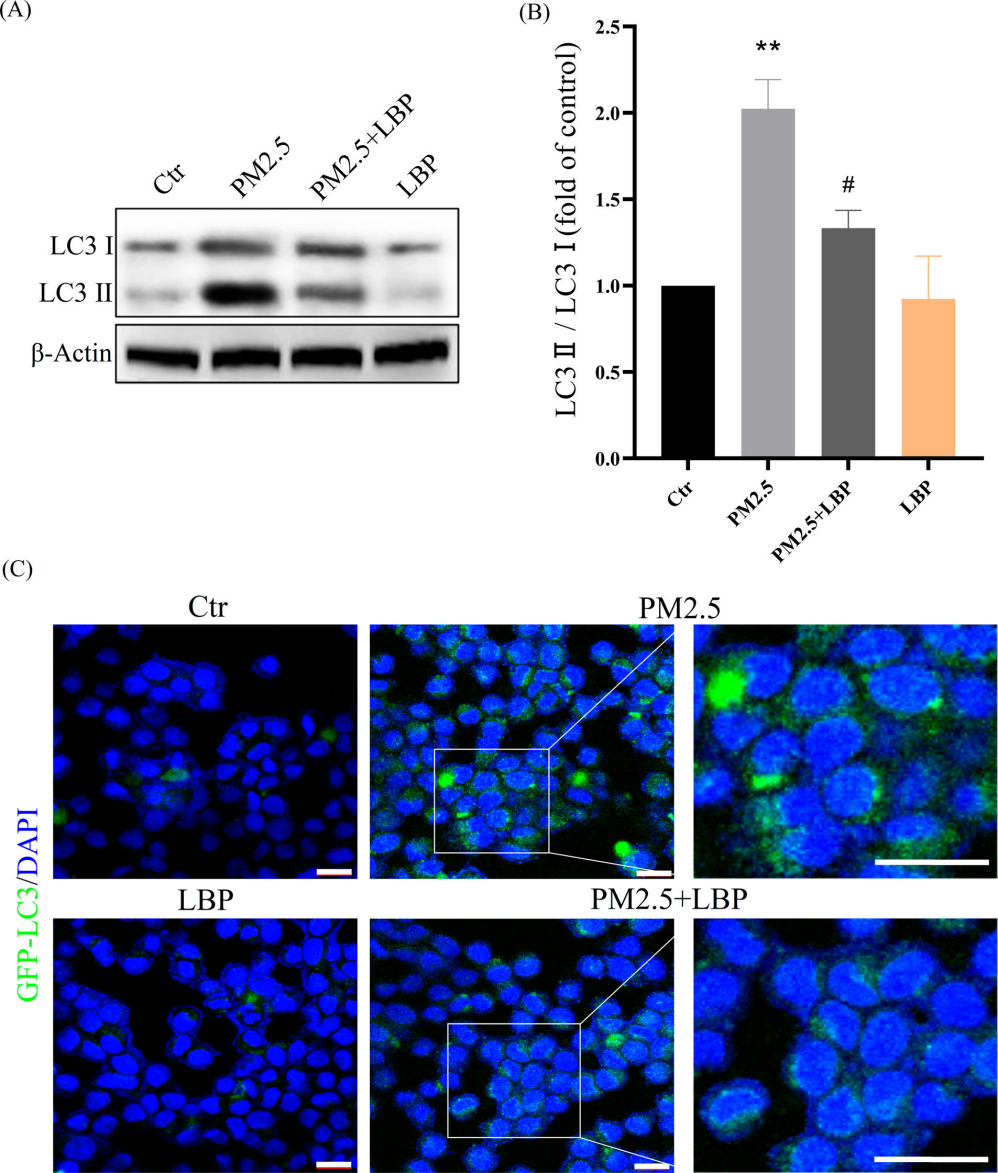
LBP inhibits PM2.5-triggered autophagy in HaCaT cells. (A) Western blot assay of the autophagy marker proteins LC3II/LC3I. (B) Statistics of LC3II/LC3I. (C) GFP-LC3 adenovirus was transfected into HaCaT cells. The fluorescent GFP-LC3 signal was used to detect autophagosomes under a confocal microscope. Scale bar = 20 μm. Values are mean ± SD. **p < 0.01 versus the control group; #*p *< 0.05 versus the PM2.5 treatment alone group.

Subsequently, we investigated the relationship between PM2.5-induced autophagy and PM2.5 cytotoxicity. HaCaT cells were pretreated with the autophagy inhibitor 3-MA and then incubated with PM2.5. The autophagy inhibitor 3-MA significantly inhibited PM2.5-induced apoptosis (Annexin V-FITC positive cells) and death (PI positive cells) (Figure 6(A)-6(C)). These results indicate that PM2.5-induced autophagy aggravates PM2.5 cytotoxicity. We further studied the effect of LBP on PM2.5-induced autophagy. The results showed that LBP inhibited PM2.5-induced conversion of LC3I to LC3II (The value of LC3II/LC3I was reduced from 2.02 ± 0.14 to 1.33 ± 0.16) (Figure 7(A) and 7(B)). Furthermore, we transfected HaCaT cells with GFP-LC3 adenovirus to study the level of intracellular autophagy. The results showed that LBP significantly reduced the level of PM2.5-induced autophagosomes (Figure 7(C)). In summary, all the evidence supports that LBP reduces PM2.5-induced cytotoxicity by inhibiting autophagy.

### 3.6. PM2.5 induces mitophagy and thus causes mitochondrial damage and apoptosis

**Figure 8.**
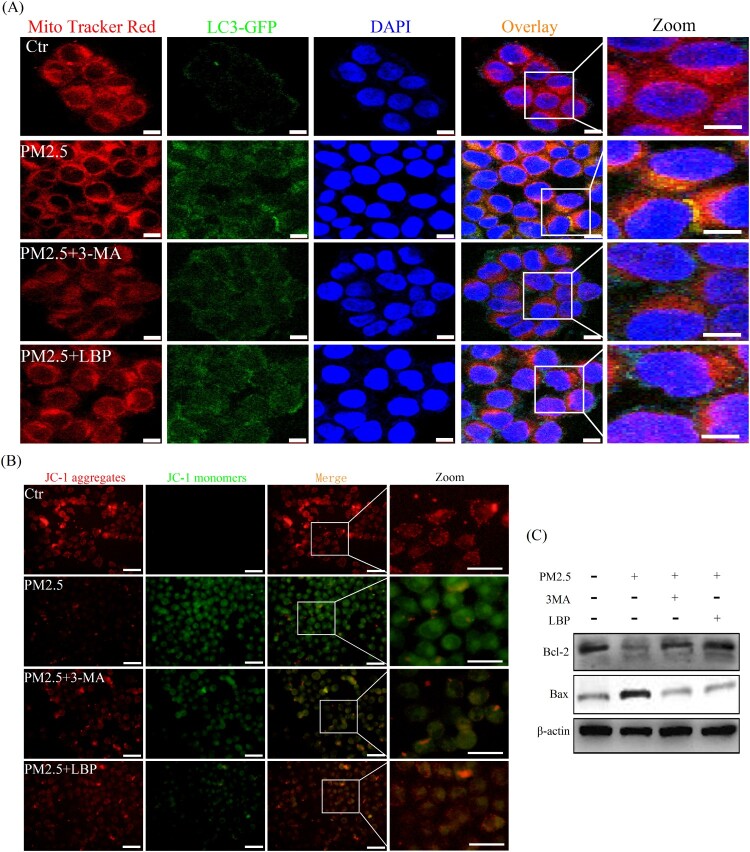
PM2.5 triggers mitophagy and activates mitochondria-related apoptosis signal Bax/Bcl-2. (A) GFP-LC3 adenovirus and Mito tracker Red were used to label autophagosomes and mitochondria in HaCaT cells to detect the process of mitochondrial autophagy (mitophagy). Scale bar = 10 μm. (B) Mitochondrial membrane potential was evaluated by JC-1 assay. Scale bar = 50 μm. (C) HaCaT cells were pretreated with 3-MA or LBP and then exposed to PM2.5 for 24 h. The expression levels of Bax and Bcl-2 was analyzed by Western blot assay. Values are mean ± SD. *p < 0.05 versus the control group; #*p *< 0.05 versus the PM2.5 treatment alone group.

According to previous studies by Fernando et al., PM2.5-induced mitochondrial damage is also the main reason for exacerbating cytotoxicity [25]. As a catabolic process, autophagy is considered to protect cells from various stress factors, and its purpose is to recycle cytoplasmic components and damaged organelles caused by various stresses [26,27]. Therefore, we further investigated the relationship between mitochondrial damage and autophagy. HaCaT cells were transfected with GFP-LC3 adenovirus for 24 h and then exposed to PM2.5 for 24 h. After that, cells were labeled with Mito tracker Red and observed under a confocal laser microscope. The results showed that PM2.5 significantly induced the fusion (yellow puncta) of mitochondria and autophagosomes, indicating that PM2.5 induces mitophagy (Figure 8(A)). Moreover, we studied the relationship between mitophagy and mitochondrial damage. The results showed that 3-MA significantly reduced PM2.5-induced mitochondrial damage in HaCaT cells (Figure 8(B)) and inhibited the activation of mitochondria-related apoptosis signal Bax/Bcl-2 (Figure 8(C)). These intracellular processes could also be reversed by LBP, further confirming the protective effect of LBP (Figure 8(A)-8(C)). In summary, mitophagy may be the main form of PM2.5-induced autophagy, which can aggravate mitochondrial damage and induce apoptosis, and LBP can protect HaCaT cells by reversing this process.

## 4. Discussion

The skin is the largest organ of the human body and the first barrier for the human body to avoid interference from the external environment [28]. With the seriousness of air pollution, our skin is more likely to be exposed to toxic substances in the air, especially PM2.5, which can cause skin aging, inflammation and even skin cancer [29–31]. It has been reported that exposure of HaCaT cells to PM2.5 can cause cellular oxidative damage, vitality reduction and apoptosis [7,8,32]. Therefore, it is urgent to seek effective drugs to prevent and treat skin damage and degeneration caused by PM2.5. LBP is a natural polysaccharide extracted from Lycium barbarum, and has been reported to have various physiological activities such as anti-inflammation, anti-aging and anti-apoptosis [33–35]. In this study, LBP pretreatment in HaCaT cells can significantly resist PM2.5-induced cytotoxicity, including restoring cell viability, ameliorating cell morphological damage, and reducing cell apoptosis (Figure 1).

Next, we studied the mechanism by which LBP protects skin cells against PM2.5-induced apoptosis and cytotoxicity. The ER is the main place for protein processing and folding in the cell, and it is the largest Ca^2+^ storage organelle in the cell [36]. When cells are stressed by external factors, the ER stress response will be activated, which may cause Ca^2+^ imbalance [37]. Previous studies have showed that PM2.5-induced ROS activate the ER stress-CHOP apoptotic signal [9], and that inhibiting the ER stress-CHOP apoptotic signal does reduce PM2.5-induced apoptosis and cytotoxicity [9,38]. In this study, LBP significantly inhibits the increase of PM2.5-triggered intracellular ROS, restores the activity of SOD and reduces the intracellular MDA level, indicating that LBP assists HaCaT cells to resist PM2.5-induced oxidative damage (Figure 2). Moreover, our results also show that LBP inhibits the expression of GRP78 and CHOP, reduces the accumulation of CHOP in the nucleus, and ameliorates the imbalance of intracellular Ca^2+^ (Figure 3). Furthermore, when the expression of GRP78 and CHOP is inhibited, the apoptosis and toxicity induced by PM2.5 are significantly reduced (Figure 4), which is consistent with the studies of Piao [38] and Molagoda [24] et al. These results confirm that LBP protects HaCaT cells from PM2.5-induced apoptosis and toxicity by regulating the ER stress-CHOP apoptosis signal.

The studies of Piao et al. have showed that PM2.5-induced ROS not only induce the ER stress response, but also activate autophagy in HaCaT cells [9]. In this study, we have found that 4-PBA inhibits the conversion of LC3I to LC3II and the formation of autophagosomes (Figure 5), which indicates that PM2.5-induced ER stress may be involved in the activation of autophagy. Autophagy is another cell self-protection mechanism that can clean up damaged organelles and recycle metabolites to maintain cell function and homeostasis [39]. However, a lot of evidence has confirmed that autophagy does not necessarily protect cells, on the contrary, it may cause negative effects and induce cell apoptosis, including our previous study on oxidative stress-mediated autophagy in ARPE-19 cells [23]. In this study, the results have shown that the autophagy inhibitor 3-MA pretreatment can reduce PM2.5-induced apoptosis and cytotoxicity (Figure 6), indicating that autophagy may be involved in inducing cell apoptosis and aggravating cytotoxicity. Furthermore, we have studied the effect of LBP on PM2.5-induced autophagy, and the results have shown that LBP also protects HaCaT cells against PM2.5-induced apoptosis and toxicity by inhibiting autophagy (Figure 7).

Different from the protective effect of PM2.5-induced autophagy in this study, the study of Dai et al. showed that autophagy could alleviate PM2.5-induced apoptosis [40], which seems to indicate that PM2.5-induced autophagy might have a different physiological function in HaCaT cells. To further study how autophagy induced by PM2.5 leads to apoptosis, we have further studied a special form of autophagy, mitophagy. Mitophagy is a cellular metabolic process, the role of which is to recover damaged mitochondria and maintain cell physiological functions [41], but continuous mitophagy may aggravate mitochondrial damage and induce cell apoptosis [42,43]. In this study, the fusion (yellow puncta) of mitochondria and autophagosomes is obviously observed in the cells treated with PM2.5, indicating that PM2.5 induces mitophagy in HaCaT cells (Figure 8(A)). Moreover, when mitophagy is inhibited by 3-MA, mitochondrial damage induced by PM2.5 is ameliorated (Figure 8(B)), and the mitochondria-related apoptosis signal Bax/Bcl-2 is also inhibited (Figure 8(C)). That is, when cells are treated with PM2.5, the pro-apoptotic protein Bax is upregulated and the anti-apoptotic protein Bcl-2 is downregulated, which are reversed by 3-MA pretreatment (Figure 8(C)). Our evidence indicates that PM2.5 induces mitophagy and thus triggers mitochondrial damage and apoptosis, which may be the reason why PM2.5-induced autophagy exacerbates apoptosis and cytotoxicity. In addition, these processes are all reversed by LBP (Figure 8), which further confirms that LBP protects HaCaT cells from PM2.5-induced apoptosis by regulating autophagy.

Although our study confirms that LBP has a protective effect on PM2.5-induced skin damage and its protective mechanisms concerning ER stress and autophagy are revealed, this study has some limitations. The relationship among intracellular ER stress, autophagy, mitochondrial damage and apoptosis is very complex and can be all triggered by PM2.5-induced oxidative stress. Therefore, there is a possibility that LBP reverses these intracellular metabolic processes by scavenging intracellular ROS. In addition, whether there are intracellular targets or specific receptors for LBP binding in regulating intracellular oxidative stress, ER stress, autophagy and apoptosis remains unclear. Metabolomics and proteomics will be used to study how LBP regulates these intracellular processes and whether there are specific receptors in the future work. It should be mentioned that 24 h treatment with PM2.5 has appeared acute cytotoxicity, but there are very few reports on the biological effects of long-term exposure to PM2.5 in HaCaT cells, which suggests that the long-term toxic effects of PM2.5 may be overlooked and should be paid attention to. Furthermore, these data and results come from *in vitro* experiments, we need to continue research in *in vivo* experiments and clinical trials in the next step.

In conclusion, we have found that PM2.5 triggers apoptosis and cytotoxicity by inducing a specific autophagy, mitophagy, and LBP can inhibit PM2.5-triggered oxidative stress, ER stress, mitochondrial damage and autophagy (mitophagy), thereby lessening oxidative damage, improving cell survival and reducing cell apoptosis. These results reveal the protective effect and mechanism of LBP against skin damage and degeneration.

## Data Availability

The data used to support the findings of this study are available from the corresponding author upon request.

## Submission declaration

We declare that its publication has been approved by all the authors.
